# The struggle against perceived negligence. A qualitative study of patients’ experiences of adverse events in Norwegian hospitals

**DOI:** 10.1186/s12913-018-3101-2

**Published:** 2018-04-25

**Authors:** Gunn Hågensen, Gudrun Nilsen, Grete Mehus, Nils Henriksen

**Affiliations:** 1 Department of Health and Care Sciences, UiT The Arctic University of Norway, Hammerfest, Norway; 2 Department of Health and Care Sciences, UiT The Arctic University of Norway, Tromsø, Norway

**Keywords:** Patient experience, Medical errors/adverse events, Open disclosure, Patient safety

## Abstract

**Background:**

Every year, 14 % of patients in Norwegian hospitals experience adverse events, which often have health-damaging consequences. The government, hospital management and health personnel attempt to minimize such events. Limited research on the first-hand experience of the patients affected is available. The aim of this study is to present patients’ perspectives of the occurrence of, disclosure of, and healthcare organizations’ responses to adverse events. Findings are discussed within a social constructivist framework and with reference to principles of open disclosure policy.

**Methods:**

This qualitative study with an explorative descriptive design included fifteen in-depth interviews with former patients recruited by the Health and Social Services ombudsmen in the two northernmost counties of Norway. Inclusion criteria were as follows: 1) experience of adverse events in connection with surgical, orthopedic or medical treatment in general hospitals; 2) men and women; 3) aged 20–70; and 4) a minimum of one year since the event occurred. Transcribed audio-recorded interviews were analyzed through qualitative content analysis.

**Results:**

The analysis revealed three main topics regarding patients’ experiences of adverse events: 1) ignored concerns or signs of complications; 2) lack of responsibility and error correction; and 3) lack of support, loyalty and learning opportunities. Patients had to struggle to demonstrate the error that had occurred and to receive the necessary treatment and monitoring in the aftermath of the events.

**Conclusions:**

Patient narratives reveal a lack of openness, care and responsibility in connection with adverse events. Conflicting power structures, attitudes and established procedures may inhibit prevention, learning and patient safety work in spite of major efforts and good intentions. Attitudes in day-to-day patient care and organizational procedures should be challenged to invite patients into open disclosure processes and include them in health and safety work to a greater extent. The study’s small sample of self-selected participants limits the generalizability of the findings, and future studies should include a larger number of patients as well as professional perspectives.

**Electronic supplementary material:**

The online version of this article (10.1186/s12913-018-3101-2) contains supplementary material, which is available to authorized users.

## Background

Illness and health problems shock people out of their natural rhythm by placing their life and health at risk [1]. People utilize health services to regain their health and welfare to the greatest extent possible and expect that their care will be performed in a safe and beneficial manner. Norway’s health service is considered high quality. However, calculations show that 13–14% of patients experience adverse events (AEs) from hospital treatments [2], and this value corresponds to international figures found in recent decades [3]. The World Health Organization (WHO) has defined an AE as “An injury related to medical management, in contrast to complications of disease. Medical management includes all aspects of care, including diagnosis and treatment, failure to diagnose or treat, and the systems and equipment used to deliver care” [4]. Patients are affected by AEs in two different ways: partly by the injury itself and partly by the hospital’s responses before, during and after the incident [5, 6].

In line with increasing international focus on the issue of AEs, Norwegian public investigations and consultations have indicated the need to develop a culture guided by principles of open disclosure in the health service. Key elements of such a culture include care for patients and their families after an injury, documentation of organizational responsibility, registration and reporting systems, investigations of undesirable incidents, systems for measuring patient safety and risk, and the involvement of patients and their families [7, 8]. Legislation has included internationally recognized principles of open disclosure [9] to ensure that injured patients receive information and help with follow-up care after incidents have occurred [10]. The Norwegian Ministry of Health and Care Services emphasizes the increasing need to account for patient perspectives in the planning and implementation of patient treatment as well as in systematic quality and safety activities [11].

Thus far, hospital responses to AEs have been deficient. An Australian study based on “100 patient stories” found a lack of open disclosure and follow-up after the occurrence of AEs [12]. These findings corroborate an American study of cancer patients’ views on apologies and open disclosure when an error occurs [13] and studies of injured patients from the UK [14, 15]. All of these results suggest a lack of recognition of patients’ need for an explanation of the event that has occurred, acceptance of responsibility, corrective apologies and initiation of measures to prevent the occurrence of similar incidents in the future. This issue is identified as the “disclosure gap” [16] or the “disclosure dilemma” [17]. Research has suggested that clinicians often avoid openness to protect their professional integrity and prevent personal consequences of an emotional, career or legal nature [17, 18]. A publication from the Harvard Medical Practice Study III concluded that “Medical-malpractice litigations infrequently compensates patients injured by medical negligence and rarely identifies, and holds providers accountable for, substandard care” [19].

To date, patient safety activities and research on AEs have largely followed a system-based and biomedical perspective [20], which has been subject to critique as a top-down approach that often excludes patient voices from patient safety research and programs [21]. Within the sociotechnical systems tradition, the Systems Engineering Initiative for Patient Safety represents a person-centered model that incorporates human factors and stands out as an alternative to more limited traditional approaches [22]. However, the authorities and health personnel appear to have prioritized clinical, efficiency-related, financial and legal perspectives [23, 24].

Scholars have critiqued these perspectives for potentially obscuring a range of social processes that affect AEs [14, 20], and have argued for closer investigation of the multiple perspectives and different versions of events that may exist [25, 26]. Ocloo states that it is time to re-identify the challenges and recognize the experiences of harmed patients as essential to patient safety efforts [14].

Clinical practice evolve through developments in medical research and interactions and negotiations between people who act within specific organizational frames that shape through professional, scientific and political discourses and the enactment of power [27–30]. These discourses and processes define the context, content of and responses to AEs and patient injuries. Extended patient safety discourses should include how professional power and control affect the articulation of patient experiences [15].

Patients possess power in the form of knowledge of their own body and health issues. However, the balance of strength and power remain askew. From a social constructivist [31] and interactionist [32] perspective, the rules of situations and settings in which social practices unfold are frames that influence actors’ behaviors. How patients perceive and interpret these frames may contribute to a broader understanding of how encounters are constructed and how power is exercised in hospitals when AEs occur.

Patient perspectives have largely been investigated using survey questionnaires with predefined questions and limited opportunities for patients to provide detailed accounts of their experiences [33], while in-depth interviews, which provide more detailed knowledge, have rarely been used [12–14]. First-hand accounts can provide authentic perspectives that illustrate patients’ experiences. The aim of this article is to illuminate conditions surrounding AEs from the patient perspective. Key aspects include how patients perceive the occurrence of events and the responses from health personnel and the health service.

## Methods

### Study design and setting

This qualitative study with an explorative descriptive design is part of an independent Ph.D. project entitled *Experiencing an Adverse Event and Life Afterwards*. The researchers have a background in nurse education, nursing and healthcare research and sociology and are affiliated with the UiT, The Arctic University of Norway. Individual open-ended interviews were considered an appropriate method for collecting data to capture complementary thick descriptions from patients about their experiences of AEs. Explorative designs allow participants an opportunity to emphasize important issues narrated from their own perspectives [34]. An in–depth interview is a *professional conversation* that seeks *deep* information and understanding of lived experiences from the interviewee’s perspective [35, 36].

### Recruitment and the sample

In Norway, The Health and Social Services ombudsman in each county can support patients and clients who experience AEs or insufficient help for their needs. The ombudsmen in the two northernmost counties Troms and Finnmark receive approximately 430 complaints related to hospital care each year [37], and were asked to assist in recruitment of study participants. As a statistical generalization was not an issue, we instructed the ombudsmen to obtain a varied sample. They performed a non-random search in their archives using the following inclusion criteria: adults between 20 and 70 years; both male and female; experience of AEs attached to surgical, orthopedic or medical treatment at general hospitals; and at least one year had passed since the event, allowing sufficient time for participants to reflect on and process the event. For qualitative in-depth studies, 10–25 interviews are considered sufficient to provide adequate data related to the research question [34].

The ombudsmen posted sixty invitation letters that included information sheets and informed consent forms that were all prearranged by the first author. The researcher did not participate in the recruitment process until the informed consent forms arrived by mail. Participants were contacted via telephone, and interviews were arranged based on their preferences.

A total of 19 former patients responded to the invitation. Two of these patients chose not to participate because they *had enough trouble* caused by the event, one had moved to a different geographical area, and one was unable to focus on the event during the interview. Thus, the study included fifteen participants, nine females and six males, ranging from 43 to 70 years old (median = 61 years). The incidents had occurred across three local hospitals and one university clinic in Northern Norway and one national hospital and one private clinic in Southern Norway. The interviews were conducted one to ten years after the AE, with an average of four years (median = 4 years). A maximum time limit was not set, as such a limit was viewed as a potential obstacle to recruiting a satisfactory number of participants. At the time of the interview, some of the informants were still undergoing treatment. All participants had experienced an injury as patients and approached the Health and Social Services ombudsman but had not necessarily claimed compensation from the Norwegian System of Patient Injury Compensation (NPE) (Table 1).

**Table 1:** Characteristics of participants, timelaps, events and compensations

Variable	*n* = 15
Gender	Female = 9
	Male = 6
Age	Lowest = 43 years
	Highest = 70 years
	Median = 61 years
Time between AE and interview	1–10 years
	Average = 4 years
	Median = 4 years
Type of event and time of occurrence/discovering	Event before treatment = 6
	Cancer diagnostic, delayed and/or missing cancer treatment:
	Breast
	Prostate
	Kidney
	Stomach/colon
	Event during/after treatment = 9
	Hip/knee prosthesis with inadequate surgery
	Surgery cheek/neck resulting nerve damage
	Incorrect anesthesia
	Incorrect medication
	Radiation injury
	Deficient stroke treatment
Application for compensation from the NPE	Yes = 10
	Received compensation = 3
	Awaiting for decision = 3
	Received refusal = 4
	Appeal against refusal = 3
	No = 5
	About to apply = 1
	Do not fulfill the criteria = 1
	Not applied = 3

### Data collection

The first author conducted individual interviews during the period from September 2013 to January 2014. Based on her experience with interview research and to position herself, she informed the participants that she had a background in nursing, that the study was independent and that she had no obligation toward the hospitals or to the Health and Social Services ombudsmen.

The conversations took place in the informants’ homes or in a desired meeting place. The first language of all participants was Norwegian, and the conversations were held in Norwegian. Each interview began with the open question: *Please tell me what happened to you ...,* and the participants were given an opportunity to speak as freely as possible to emphasize their own reflections and understandings. The researcher took a listening role and asked follow-up questions to increase the richness and depth of the stories [34]. A thematic interview guide formulated from findings of previous research [5, 6, 12–14] was used as a supporting document (see Additional file 1). ZxXThis guide served to ensure coverage of main themes across patient stories and to obtain more details about statements or topics if necessary [34]. Examples of follow-up questions include: *What do you mean by that? Can you explain more about that?* Side notes were written during the interviews. As no substantial new information appeared during the last interviews, the sample size was considered adequate for research purposes. The interviews lasted 45–150 min, were recorded as sound files and were transcribed to text by the first author. Expressions such as silences, sighs, laughter, crying, etc. were noted because they may influence the underlying meaning [38]. Eight of the participants released their discharge reports as supporting material.

### Ethical considerations

Ethical considerations corresponding with the Helsinki declaration and national research regulations were made throughout the entire project [39, 40]. After receiving oral and written information about the design and goals of the study, all participants provided their voluntary approval for participation and publication by signing an informed consent form. The participants were informed about their right to withdraw at any time, without stating a reason, and were guaranteed confidentiality and the anonymous presentation of findings. Fictitious names are used in the presentation of the results.

### Data analysis

In qualitative research, analysis involves hermeneutic processes in which a pre-understanding derived from personal experience, former research and theoretical perspectives meets data and produces a deeper understanding and new concepts [38, 41]. All text from the interviews (approximately 141,600 words) was subjected to the analysis, which was inspired by Graneheim and Lundman’s inductive model of qualitative content analysis [38] and supported by Malterud’s approach [34]. Our analysis addressed the manifest content aspects and describes the visible, obvious components in the texts as well as the latent underlying meaning based on interpretation. The texts were reread a number of times (GH and GN all interviews; NH 5 interviews) to gain an overall understanding and to generate preliminary categories.

Next, we made thematic categories and categorized the material in the texts that addressed the experiences related to the event itself. We then analyzed this material closely for the current paper. Statements related to the same central meaning were converted into condensed meaning units, which were coded and further interpreted and categorized as sub-topics and finally collected into main topics without use of qualitative software. The manifest content addressed the event that occurred, where and when it occurred, who was involved, how it was handled, and the patients’ perception of the event. Additionally, latent content, e.g. *cover up*, arose ‘unexpectedly’ or inductively from interview statements and was subject to interpretation. Coding and interpretations were checked against original transcripts. An example of this analysis is provided in Table 2.

**Table 2:** Example of analysis

Meaning units	Condensed meaning unit	Interpretation of the underlying meaning	Subtopic	Main topic
“I didn’t like that lump and I felt strongly that something wasn’t right ... The lump was visible and painful, I went to the doctor several times and tried to speak up ..., but mammography and tissue samples had been taken and the specialist at the hospital had signed them as normal.”	Strong concern something is wrong. The lump is visible and painful. Tries to get help, but is rejected	Tries to take care of own body and health, but feels rejected	Being rejected and not heard “to be ignored”	Ignored concern or signs of complication
“I was kind of naïve and believed that when the error first was proven, they would get the grip of things and act very fast, but that certainly did not happen. I had to call, and call and call…That was the worst with the situation”	Expects the hospital to get the grip of the proven error fast. Disappointed they did not act rapid on fault correction The struggle for treatment makes the situation worse	Missed expectations of hospital responsibility and fault correction	Responsibility Fault correction	Lack of responsibility and fault correction
“The doctor I spoke to said that the first doctor had made an incorrect assessment, but that the system then got involved. A cover-up begins, and there are rules about what they can say and what they can do. Anyway, I am glad he was honest enough to say that. It helps a little.”	Feeling confirmed something is wrong. The systems with cover up takes over. Is good to know about the error anyway. Limited support	Needs support, but professional loyalty is more important	Support Professional loyalty	Lack of support, loyalty and learning

Trustworthiness of the data was achieved through reading the manuscripts multiple times and discussing themes in a series of meetings, and the three researchers’ independent generation of topics and themes. Discrepancies were resolved through several discussions until consensus was reached. The authors had different levels of experience with AEs from their professional and personal backgrounds. Reflexivity, practiced by noting biases and prior expectations, was an important piece of the analysis process to elicit the new understandings. The discharge reports were analyzed in light of whether the events were described and how they were presented. All the authors discussed the topics in light of relevant theory and achieved overall agreement. The following main topics emerged from the data (Table 3): ignored concerns or signs of complication; lack of responsibility and error correction; and lack of support, loyalty and learning. All study participants (*n* = 15) reported experiences that related to each of the three themes.

**Table 3:** Overview of theme, main topic and subtopic

Theme	The struggle against perceived negligence		
Main topic	Ignored concerns and signs of complications	Lack of responsibility and fault correction	Lack of support, loyalty and learning
Sub-topic	The feeling of something wrong To speak up Being ignored/ rejected/ not heard Falling out of the system	Feeling “life is at stake” Disclosure, explanation, apology Feeling avoided Waiting time Responsibility Fault correction	The need of support and understanding Professional loyalty/ “Cover up” system Possibility for learning

## Results

### Descriptions of events

The findings concern AEs in various stages of the treatment chain and cover a wide range of basic illnesses and degrees of severity at the time of the event. A common feature among the fifteen study participants was that they experienced that *something* or *someone* had failed. The accounts represent many common denominators with regard to the overall sequence of events; reaction patterns; and patients’ perceptions of health personnel, the system, the care provided, and follow-up afterwards. Failures and defects are found in medical treatment but also in communication, information and documentation. Six of the events were related to failures and defects before treatment began. In these cases, the patients received a delayed or incorrect cancer diagnosis, which postponed the start of cancer treatment.

Various forms of cancer were represented: breast, prostate, kidney and stomach/colon. The informants assessed the causes of the events as follows: incorrect medical assessment of clinical signs and symptoms, failure of diagnostic tests, notices of appointments that were sent incorrectly or were missing or test results/referrals that were put aside for later examination but were not assessed. According to the informants, the delays resulted in an increased spread of cancer; more severe complications, resulting in more complicated treatment regimens; and mental stress. The informants mentioned the stress involved in getting cancer but stated that the most difficult aspect was the perceived incorrect or deficient treatment, which they viewed as a sign that their lives and health were not sufficiently valued.

The other nine events represent experiences related to orthopedic conditions, such as hip or knee interventions; surgery on the cheek/neck; incorrect anesthesia; incorrect medication; radiation injury; and deficient stroke treatment.

The events include errors/failures that were discovered while the patient was in hospital as well as errors/serious complications that became evident after discharge. Several of the informants believed that medical assessments, test analyses or organizational/administrative systems did not function properly. According to the interviewee’s, the events resulted in functional impairment requiring further surgery or treatment, difficult rehabilitation, organs that failed, pain and a more complicated sequence of treatment.

### Ignored concerns or signs of complications

All the informants understood that hospital treatment involves a certain degree of risk. They stated that information about operations, other treatments and delayed diagnosis was presented in a standardized and *everyday* manner. When an undesirable event occurred, the health personnel did not appear to relate to the event or come to grips with the problem that the patient experienced Health personnel were perceived as trivializing, rejecting, skeptical or doubtful.

Most of the respondents described a powerful inner concern that something was wrong but felt as though they were ignored or overlooked when they mentioned their concern. In the case of diagnostic errors, the concern was related to bodily symptoms or a long wait for or absence of further tests and treatment. A woman with a delayed diagnosis of breast cancer explained:

Eva: “*I didn’t like that lump, and I felt strongly that something wasn’t right ... The lump was visible and painful. I went to the doctor several times and tried to speak up ..., but mammography and tissue samples had been taken, and the specialist at the hospital had signed them as normal.”*

It was later proven that an error had been committed with the samples and that the cancer had been discovered many months earlier. The test results had rested in a pile at the hospital for new assessment, but a review was not performed. Positive test results were found coincidentally.

The events that became evident during the hospital stay or after returning home concerned symptoms of errors or of complications assessed as normal issues or symptoms that would disappear over time.

Peter: *“I asked how the operation had gone, and the doctor said that it had been a little more complicated than first envisioned, but everything had gone well. I had pains when discharged, but I could still manage to move my arm. At home, I suddenly could not hold my arm out. I contacted the doctor at the hospital; he said it was completely normal and I could relax. It would get better again. But it certainly did not: it got worse and worse. I spoke to the hospital again but got the same answer ...”*

After a long struggle, neurological examinations found nerve damage that resulted in permanent pain, paralysis and loss of function.

When errors became visible, health personnel appeared unwilling to talk about the errors, support the patients during a difficult situation, or help with further follow-up and treatment.

### Lack of responsibility and error correction

The informants reported their experiences of being in the middle of situations that were decisive for life and health. They had expectations that any damages and errors would be rapidly limited and corrected, but they were disappointed. Few of the informants received an apology or detailed explanation of the event that had occurred, and none of them had a meeting with those involved to clarify the situation afterwards. The two informants who received an apology and explanation viewed them as noncommittal because they were not invited to share their experiences with the staff in general. Most informants perceived that hospital staff denied responsibility by avoiding dialogue and not providing suggestions for correction and damage limitation. The informants clearly expressed their disappointment.

Kari: *“The doctor held his head up and walked straight past me; I know he saw me - that was a really unpleasant feeling.”*

Tone: *“I had gotten much worse, but the doctor just turned around and walked out. And that was that. It was this denial of responsibility; that you were not allowed to talk about it like ... it would have meant a lot to me to have a good discussion with someone...”*

The accounts show that it was essentially the patients’ responsibility to prove the AE. They encountered a hospital culture that normalized and trivialized AEs by referring to the fact that current procedures and rules had been followed and/or that tests and examinations showed normal findings.

Bjørn: *“Not very much I can do when the doctor says it looks fine. You have to be quite active and more or less healthy to be able to keep up with all this. I don’t know ... you feel almost like a scoundrel. Feel that you are being met with doubt the whole time.”*

This was experienced as a challenge in that patients can refer to only unexpected symptoms and feelings. Patients experiencing pain, discomfort and vulnerability must struggle to ensure that their condition is re-assessed. In Camilla’s case, after a hip operation, the prosthesis came out of joint. She heard a *click*, which was followed by serious pains and mobility problems. The staff thought that the pains were normal postoperative pains, and the patient had to make a fuss to get a new X-ray, which confirmed the luxation. She then had to wait four painful days for reoperation, which revealed a splinter of bone that had remained in the hip joint. Other informants experienced similar difficulties with circumstantiation and clinicians who they perceived as rejecting and arrogant:

Nina: *“I could see for myself that the muscle was just hanging, but they said it would get better.” “He (the doctor) said that everything was perfect without touching me. I couldn’t understand how ... he had just done perfect work.”*

These deficiencies reinforced the informants’ feelings of discouragement, vulnerability, pain and bitterness. The interviewees perceived that those who were responsible did not wish to accept the consequences of their decisions and actions, while the patients had to struggle to receive further help and treatment. The informants felt that they were not prioritized and that the time between the discovery of the error and the start of treatment could be long.

Peter: *“Everyone can be unlucky, but can’t they just be nice enough to say so. Say ‘we’ll help you’ -why can’t they do that?”*

Responsibilities regarding follow-up appeared to be unclear, and the informants stated that they were placed on new waiting lists where they received little or no information about what was to be done and when.

Eva: *“That was when the struggle began, and that was the worst. I was naive enough to think that once an error had been discovered then things would move quickly. Nothing could be done about what had happened, but they could come to grips with things quickly, but that didn’t happen ... I had to fight my way to treatment, pure and simple. I had to stay on the phone and ring, and ring, and ring ...”*

### Lack of support, loyalty and learning

All the informants felt alone with their problems. Admitted patients found that hospital personnel rarely noticed their need for physical or mental support.

Kari: *“There has been no real follow-up with me as a person, and I don’t feel that I have had any support ...”*

Nurses and other carers showed understanding related to the performance of concrete tasks such as pain relief, showering or toilet visits but were otherwise perceived to be silent and absent. However, there were references to nurses who expressed understanding and recommended that the patient seek help from the patient and services ombudsman or report the incident as a patient injury. Informants described physiotherapists as one professional group that was supportive and asked questions about the patients’ progress and if they could help in any way.

The informants described how errors and defects in case notes made the course of further treatment difficult. Hospital doctors, other health personnel and GPs did not have knowledge about earlier events or complications due to a lack of written documentation in patient records. Some of these professionals notice problems and support the patient, while others are more concerned about documentation in the medical records before they refer patients to new examinations, check-ups and rehabilitation. The released discharge reports confirm the lack of descriptions of events and complications. When new health professionals must refer to incomplete written records, it is difficult for the patient to gain acceptance of his or her version and participate in decision making that could correct the error or prevent a new error from occurring. Thus, reoperations or corrective treatments are viewed as *new* treatments without considering the situation as a whole. Informants perceived this view as problematic and expressed that clinicians and administrative systems should be aware of the event, facilitate better follow-up, and be more attentive to patient needs in the situation.

Several of the informants spoke of specialist assessments and second opinions that finally supported their assertion that an error had occurred. The feeling of being seen and cared for as a person was of great significance. Kari explained this when describing the surgeon who performed the reoperation.

Kari: *“He has followed up, telephoned me and given me very good information. This is reassuring, and it is directed at me as a person, not just two knees.”*

However, informants also found that verbal feedback did not necessarily correspond with written statements in the medical records.

Bjørn: *“He actually saw what was wrong with me straight away. It was good to have it confirmed ... but he did not want to stab a colleague in his back, which is why the report is as it was. However, he would follow my case, and he was one hundred per cent behind me. Therefore, I imagine he has something of a bad conscience. This hierarchy is like nothing else in society. They can’t just sit and cover each other.”*

Mari: *“The doctor I spoke to said that the first doctor had made an incorrect assessment but that the system then got involved. A cover-up begins, and there are rules about what they can say and what they can do. Anyway, I am glad he was honest enough to say that. It helps a little.”*

The informants appeared to be strongly affected by the events and their impact on their lives. At the same time, they believed that health personnel and the health service at both the individual and system levels should learn from the events and prevent similar events in the future. They interpreted the experience of a lack of recognition, disclosure and understanding to imply that learning is hindered when events are trivialized to such an extent.

## Discussion

### The struggle against perceived negligence

The purpose of this study was to illuminate patients’ experiences of AEs as bottom-up inputs in patient safety work. Our sample represents a variety of diagnoses and conditions. The fact that all the “before treatment” cases in our study involved cancer, in contrast to only one of the during/after treatment cases, was somewhat surprising. This may be due to the sample composition. However, we are unable to evaluate this issue further at this time. One major finding concerns patients’ struggle against perceived negligence by clinicians and hospitals when they present their anxiety and concerns or point out errors and deficiencies. In summary, these reports describe the clinicians’ and health service’s avoidance or lack of response, signs of denial of responsibility and use of loyalty systems to largely support and protect each other. In these cases, patients are shocked by illness [1], and this shock may double due to injury or even triple due to the lack of adequate follow-up and treatment. The descriptions reveal potential barriers to openness and indicate that these patients were not invited into processes of open disclosure. This finding is in line with results reported by Iedema et al. [12], Ocloo [14] and Mazor et al. [13], who found that patients clearly expressed that a patient-centered and respectful dialogue to promote healing, learning and safety should follow the disclosure of an AE. In our study, informants’ descriptions also revealed a practice that was not in line with Norwegian governmental policy statements regarding the promotion of trust and openness in patient safety work [7].

However, some informants reported positive experiences of health personnel who acknowledged their experiences, offered help related to their condition and supported them in reporting the incident.

On the other hand, health personnel have been described as the *second victims* and also experience great stress from AEs; these factors must be taken into consideration [42]. Furthermore, individual clinicians may lack communication skills and may generally find it easier to avoid difficult conversations [18]. Several informants referred to their perceptions of doctors’ reluctance to criticize colleagues openly. This reluctance could connect to the Medical Associations’ ethical rules [43] of the handling of such events between colleagues but could also indicate that professional codes of collegiality and loyalty exist within the medical profession and potentially hamper an open disclosure process [17, 18].

At a more general level, the breaches may represent more or less latent and unrecognized driving forces against defining errors and serious complications in hospitals. Hospitals work hard to avoid negative figures in reports and/or to maintain their reputation and income; paradoxically, such efforts may tend to counteract openness about undesirable circumstances [17]. However, we have no data to evaluate the extent to which hospitals used the errors narrated by study participants in learning processes to improve patient safety. Nevertheless, our findings may be interpreted as signs of professional and organizational cultures that do little to communicate errors to patients and to include them in learning processes at any organizational level, which potentially impedes open disclosure and even hampers learning from AEs as outlined in high-quality guidelines, e.g. guidelines from Harvard hospitals [9]. This could contribute to the understanding of why patients struggle to obtain evidence that recognizes the event and promotes further support.

### The discursive power

The participants’ perception of being ignored and that the definition of events was not in line with the personal consequences that they experienced, was problematic. Our findings indicate that practitioners and hospitals have discursive power in the form of *expert knowledge*, which determines and limits the prevailing definition of the truth in the situation [27]. In general, professional knowledge and the hospital environment give health personnel an authority to assess the best course of action in any patient situation. Participants reported lack of acknowledgement of their condition in the encounter with the experts. This finding corroborate results from Eriksson and Svedlund who studied patients’ dissatisfaction with hospital care [44]. The results further shows that patients did not experience to be part of assessment processes or in the social construction of categories that defined what was considered normal in the patient’s situation. Sharpe and Faden argue that the definition of medical injury is overly one-sided and tends to reflect a narrow clinical understanding that excludes non-clinical perspectives and outcomes that the patients experience as damaging [45]. In this sense, the understanding of AEs may be selective and contribute to disciplinary actions and stigmatization [30]. Events described as *foreseeable complications, misunderstandings, organizational failure* etc. place the responsibility outside the potential control of the health personnel and attribute the events to chance, natural surgical risk or patient factors.

Some informants reported feeling uneasy when asking questions. Patients who speak up and ask questions may thus be perceived as extra concerned, having a negative attitude, being bothersome, difficult to treat etc. When problem descriptions take little account of patient perspectives, there is a risk that general intentions about increased patient involvement, empowerment, openness and AE reduction are not spread downwards through the organization and do not make contact with the prevailing values of the professional and organizational cultures. In practice, this may imply that a number of patients do not receive the treatment and follow-up that they need and begin a difficult process to ensure that their condition is acknowledged. The patients in our study were not involved in designing measures to avoid incidents, and to their knowledge, their experiences were not pursued for subsequent learning. Thus, intentions regarding patient-centered care and patient participation in the development of safety efforts appeared to be practiced to a limited extent in the reported cases.

### Patient safety within fixed frames

Patterns of action that replicate many times in organizations like hospitals, become typified and institutionalized as permanent attitudes, actions and methods that tend to be taken as a given [31, 32]. Within these frames, social practice unfolds. In this way, awareness in the everyday understanding of patient treatment has become routine. This issue not only applies to individual errors or deficiencies related to everyday performance, but may also represent practice cultures in which the exclusion of patients and events becomes routine and maintained by the prevailing knowledge regimes and professional hierarchies. Changing these regimes requires increased awareness, not only in terms of the authorities’ intentions to increase patient safety but also in terms of the inclusion of both personnel and patients at all levels of professional and organizational cultures [31].

Learning processes can be inhibited if the goal, in this case, the reduction of AEs and improved follow-up with patients, does not coincide with other goals, such as maintaining professional autonomy, promoting the individual clinician’s career opportunities or earning the status of the professional center or hospital with the fewest reported errors. Argyris and Schӧn [46] refer to this dilemma as the differences between *espoused theory* and *theory-in-use*, where ideals and reality do not concur. Changing fixed attitudes and patterns requires obtaining information that includes all relevant perspectives, weighing alternative knowledge-based actions, continuously assessing the consequences of the action taken and questioning whether the action is congruent with governing values [46].

The study results show that patient experiences may serve to identify barriers to patient safety work that are necessary to overcome to prevent future AEs.

### Strengths and limitations

The data are from interviews with a small, selected group of patients who had the energy and resources to contact the Health and Social Services ombudsman for assistance. Thus, the findings cannot be generalized to the entire population of patients who experience AEs in Norwegian hospitals. Personal stories might also be selective, exposed to recall bias and affected by a wish to narrate only aspects that favor the quest for some kind of compensation following an AE. In addition, the relatively long time span between the incidents and the interview may have provided informants with new contexts of interpretation due to their personal biography and the increased public attention toward patient safety issues. On the other hand, an expanded time frame may contribute to greater richness in terms of reflections on the event. Overall, the patient stories bear significant similarities and appear to be consistent across all subject areas and diagnoses, across a wide range of institutional settings, and regardless of whether the persons had applied for or received compensation. This consistency should contribute to the trustworthiness of results and indicates that the stories reveal important aspects of a widespread deep structure within hospitals that inhibits the realization of principles of open disclosure. Another important limitation is that the study does not include clinicians or health service providers’ perspectives of the events. Some of the events may expose grey areas between complications and AEs. Nevertheless, experiences of a struggle against perceived negligence are present across all stories.

### Implications

Patients are in need of emphatic and professional acceptance of responsibility when an error occurs. Health personnel must listen to the patient and bear in mind that communication and open dialogue have great implications for the person’s evaluation of the event and future life and health. This qualitative study may promote reflection among care providers irrespective of professional area, hospital management and government. Acknowledging that patients are co-producers with important knowledge about their health situation is essential to the development of safer treatment. Future research should include a larger number of patients as well as organizational and professional perspectives. The impact of AEs on the daily lives of patients and their families should also be studied.

## Conclusions

This small-scale, qualitative study shows that some patients experience a lack of care, openness and acceptance of responsibility when AEs occur. They perceived that their perspective was largely ignored. We have no data to evaluate whether their cases are used as input for learning and development in patient safety work. However, open disclosure guidelines advocate for the inclusion of patients at early stages and throughout the process. Our results indicate that the full potential of such learning and development is not realized in hospital units. Considerable cultural and attitudinal changes in day-to-day patient care are necessary. Patients exposed to AEs should be invited into open disclosure processes and included as fellow architects of their own health and safety.

## Additional file

Additional file 1: Interview guide. (DOCX 16 kb)

## Abbreviations

AE: Adverse events
NPE: Norwegian System of Patient Injury Compensation
NSD: The Norwegian Social Science Data Services
REK: Norwegian regulations for ethical research practice.
