# Correction to: The struggle against perceived negligence. A qualitative study of patients’ experiences of adverse events in Norwegian hospitals

**DOI:** 10.1186/s12913-019-3974-8

**Published:** 2019-03-14

**Authors:** Gunn Hågensen, Gudrun Nilsen, Grete Mehus, Nils Henriksen

**Affiliations:** Department of Health and Care Sciences, UiT The Arctic University of Norway, Hammerfest, Norway

**Correction to: BMC Health Services Research (2018)**

**https://doi.org/10.1186/s12913-018-3101-2**

In the original publication of this article [1], the corresponding email was changed to ghagensen@gmail.com.
