# Supplementary material for: The struggle against perceived negligence. A qualitative study of patients’ experiences of adverse events in Norwegian hospitals
Source: BMC Health Serv Res. 2018 Apr 25;18:302. doi: 10.1186/s12913-018-3101-2 (PMC5921559; doi:10.1186/s12913-018-3101-2)
Supplement: Supplementary file 1 — Interview guide. (DOCX 16 kb) [file 12913_2018_3101_MOESM1_ESM.docx]

**Interview Guide:**

**Issue:** Patient experiences with adverse events.

Before the interview begins, the researcher gives a presentation of the project, repeats the content of the written information sheet and consent statement.

During the conversation, the participant should be encouraged to talk as freely as possible about the adverse event. The interview guide serves as support. The researcher holds a listening role and asks follow-up questions to obtain more information about statements or if necessary to introduce a new topic from the interview guide. The focus is upon the participant’s story and the interview further elaborates issues that the participant brings out.

**Introduction**

**Main question:** «How is it to experience an adverse event in hospital, and what challenges does it bring along to the life afterwards?»

**Opening phrase:** Please, tell me what happened to you…

**Part 1. The adverse event and the hospital stay/contact**

Describe the event and explain how you experienced it…

How did the health personnel and hospital handle the event?

- Did you get information of what had happened – in what way and what kind?
- Degree of openness/transparency
- Apology, in what way and from whom?
- Help and support with follow up afterwards. Physical and psychological, new treatments if necessary and recovery process.

**Part 2. The life afterwards**

In what way did the event influent on your further life, in short and longer term?

- Health conditions
- Daily life
- Family life
- Working life
- Economy
- Other issues

**Part 3. Your meeting with and interaction with the health services afterwards.**

How has your contact with the health services been afterwards?

Did the event affect your trust to the service or to the health personnel? If it has, in what way?

Please, tell more about:

- communication
- interaction
- follow- up

**Closing**

Is there anything you think could have prevented the adverse event to happen?

What do you consider as important to attend to when someone experience an adverse event?

Is there anything you would like to add that we have not covered?

Thank you for the participation!
